# Nanomaterial Effects on Photosynthesis in C3 and C4 Crops: A Comparative Review and Tentative Design Principles

**DOI:** 10.3390/nano16211437

**Published:** 2026-11-06

**Authors:** Yihan Wu, Jingyi Zhang, Yukui Rui

**Affiliations:** 1 State Key Laboratory of Nutrient Use and Management, Beijing Key Laboratory of Farmland Soil Pollution Prevention and Remediation, College of Resources and Environmental Sciences, China Agricultural University, Beijing 100193, China; wuyihan0115@163.com (Y.W.);; 2 Professor Workstation in Shanghe County, China Agricultural University, Jinan 251600, China; 3 Professor Workstation in Wuqiang County, China Agricultural University, Hengshui 053300, China; 4 Professor Workstation of Beijing Yuzeshengda Technology Development Co., Ltd., Huairou District, China Agricultural University, Beijing 101400, China

**Keywords:** nanomaterials, photosynthesis, C3 crops, C4 crops, nano-fertilizers, hormesis, design principles

## Abstract

Engineered nanomaterials (ENMs) can regulate crop photosynthesis, but the same nanoparticle may stimulate or inhibit carbon assimilation depending on dose, material properties, delivery route, and crop species. Existing reviews largely treat crops as interchangeable and report rather than predict effects. This review organizes the evidence around the C3/C4 photosynthetic distinction. We contrast C3 and C4 architectures, and CO_2_-limited, photorespiration-prone C3 leaves versus Kranz-type, CO_2_-concentrating C4 leaves and synthesize how metal-based nanoparticles, carbon dots, and nanofertilizers affect light harvesting, electron transport, Calvin-cycle enzymes, stomatal behavior, and source–sink relations, separating direct interactions from nutrient-, water-, and redox-mediated indirect effects. Head-to-head C3–C4 trials remain uncommon, yet those that exist point the same way: the two groups move in one direction within a given experiment, differing only in how large the movement is. The administered dose is also a poor guide to the dose that actually reaches a chloroplast. As the dose rises, plants pass through a sequence—no response, adaptive signaling, physiological stress, and finally harm. Whether the outcome is positive or negative is governed by material and dose rather than by photosynthetic type; the latter changes only the strength of the response. These observations suggest tentative rules: C3 crops are best served by ENMs aimed at CO_2_ supply and photorespiration, whereas C4 crops benefit more from ENMs targeting electron transport, sink strength, and stress. The way forward lies not in inventing still more materials but in standardized, physiology-informed designs that let the field predict responses instead of merely cataloging them.

## 1. Introduction

Photosynthesis is the largest of the global carbon fluxes, with terrestrial gross primary production estimated at approximately 123 ± 8 petagrams of carbon per year from observation-based eddy-covariance networks and at approximately 147 petagrams per year (95% interval 131–163) by recent satellite-based near-infrared-reflectance scaling [1,2], and it supplies nearly all of the assimilates on which human nutrition depends [3], yet its efficiency in most field crops remains far below the theoretical maximum [4] and improving this efficiency has been identified as a major underused lever for raising yield potential [5]. Closing part of this gap is one of the most consequential challenges in agricultural research, and nanotechnology has been repeatedly proposed as a route toward it. Engineered nanomaterials can be applied as foliar sprays, soil amendments, seed coatings, or root drenches. They have been reported to alter pigment content, photosystem activity, gas exchange, antioxidant status, and ultimately yield in a wide range of species [6,7,8]. How these nano-enabled products measure up against their conventional counterparts has been examined in detail [9]. The breadth of these claims is unusual: positive photosynthetic effects have been described for carbon dots [10,11,12], TiO_2_ [13,14,15], ZnO [16,17,18], CeO_2_ [19,20], iron oxides [21,22], and other compositions, while equally well-documented studies report inhibition, null effects, or species-specific toxicity [23,24,25,26].

This body of work is not easy to turn into agronomic advice. Most studies read as isolated case studies: the nanomaterial, dose, application route, and measured endpoint all differ, so no quantitative pattern carries across studies [27]. A further complication is how plants are classified—research is organized by species rather than by photosynthetic type, even though C3 and C4 metabolism place very different limits on water use, CO_2_-concentrating mechanisms, and canopy photosynthesis [28]. Dose–response information is also commonly flattened into a single “beneficial or toxic” label, discarding the information needed to define safe application windows [14,29]. These topics have not gone unreviewed. Recent surveys cover nanofertilizers and photosynthetic efficiency [30], carbon nanomaterials and plant growth [31], nanobionics in crop production [32], nanofertilizer effects on yield and quality [33,34], nanoparticles under drought stress [35], and the use of nanomaterials for stress resilience [36,37]. Others address the physiology and environmental resilience of nano-enabled crops [38], approaches to mitigating drought stress [39], the applications, toxicity, and trends of nanoparticles in agroindustry [40], and nano-enabled strategies for crop nutrition and protection [41]. Yet none of them treats the C3–C4 dichotomy as an explanatory variable, or pulls the scattered dose–response results together into design principles that could guide actual applications.

The objective of this review is therefore threefold. First, we establish a corrected physiological baseline by describing the distinct architectures of C3 and C4 photosynthesis and by explaining why these differences should condition the design and interpretation of nanomaterial experiments. Second, we review the mechanisms by which ENMs affect both the light and dark reactions of photosynthesis, focusing on carbon dots, whose uses as photonic nanomaterials are especially broad [10,11,42,43]. Third, we organize the dose–response evidence into a conceptual photosynthetic dose–response framework that distinguishes a no-response zone, an adaptive-signaling zone, a physiological-stress zone, and a harmful zone, and we discuss the methodological and regulatory barriers that currently prevent field-scale application [26,44,45]. Throughout, we mark the points where evidence is contested or simply missing rather than polishing them flat, because nano-enabled agriculture can only be credible if its unknowns are visible.

Previous reviews span much of this ground, from uptake and toxicity to nanofertilizer performance and the behavior of individual material classes. Almost none of them, however, separate crops by species. One plant is treated as a substitute for another, and effects are narrated instead of forecast. We begin instead from the C3/C4 divide and the specific physiological step that limits each type. From there, we collect the handful of matched C3/C4 comparisons that genuinely inform the question, taking care to separate what has been demonstrated from what is merely inferred. The patterns that remain can be written as design principles specific enough to test and then revise. The value of this approach is not a new material but a more reliable way of reading the existing evidence, distinguishing stimulation from recovery, measured mechanism from proposed mechanism, and nominal dose from the dose that reaches the chloroplast, and thereby shifting the field from reporting isolated responses toward anticipating to which nanomaterial a given crop will respond, at which step, and under which conditions.

## 2. Photosynthetic Architectures of C3 and C4 Crops

**Figure 1:**
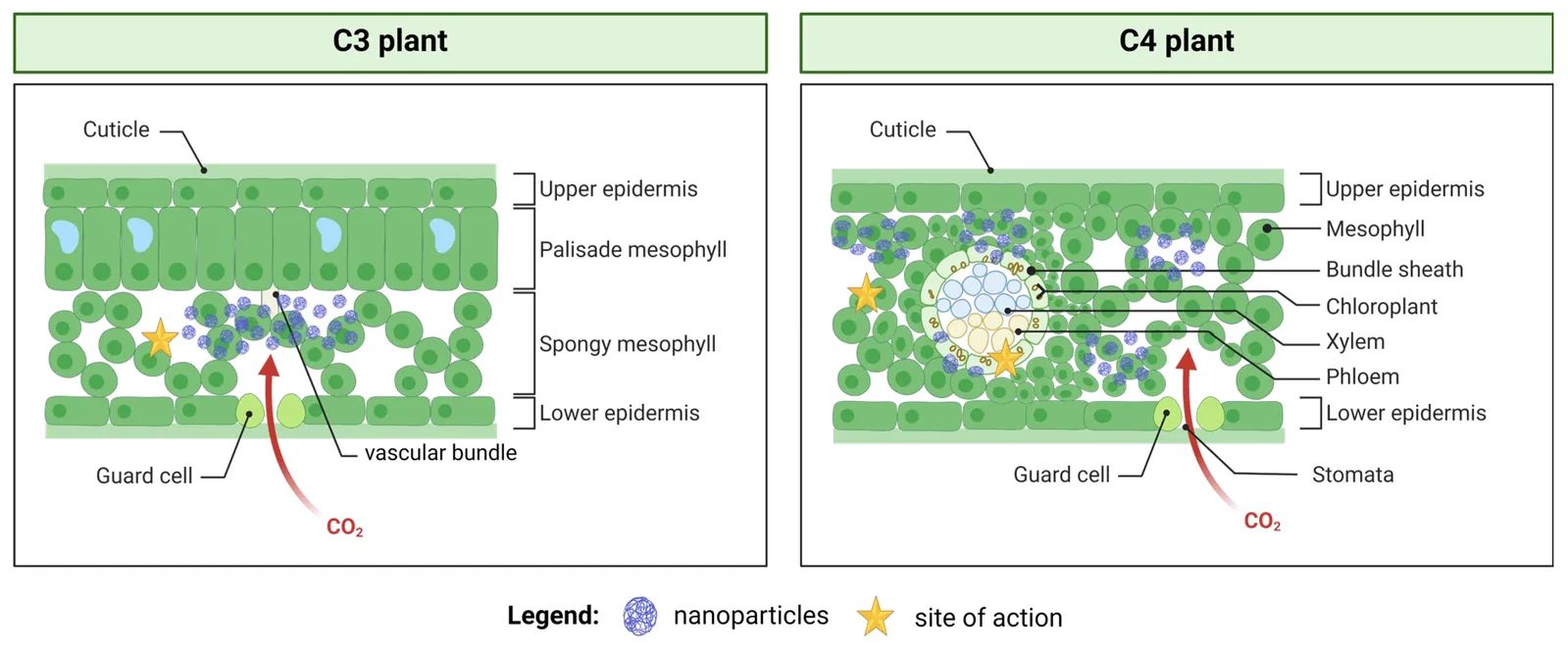
Comparative leaf anatomy and nanomaterial action sites in C3 and C4 plants. (**Left**) C3 leaf cross-section showing differentiated palisade and spongy mesophyll and a simple vascular bundle; nanoparticles enter through the stomata and act on mesophyll chloroplasts. (**Right**) C4 leaf cross-section showing Kranz anatomy, with bundle-sheath cells surrounding the vascular bundle (xylem and phloem) and mesophyll cells arranged radially; nanoparticles act on both mesophyll and bundle-sheath chloroplasts. The legend identifies nanoparticles and sites of action. Created in BioRender: https://BioRender.com/mu282bu (accessed on 26 October 2026).

A correct understanding of the physiological differences between C3 and C4 plants is a prerequisite for interpreting nanomaterial effects, yet this baseline is often treated imprecisely in the nano-agriculture literature. In C3 plants—rice (*Oryza sativa*), wheat (*Triticum aestivum*), soybean (*Glycine max*), and most temperate crops—CO_2_ is fixed directly by Rubisco in mesophyll chloroplasts; because Rubisco also accepts O_2_, photorespiration occurs when CO_2_ at the active site is low relative to O_2_ [5], and CO_2_ diffusion into the leaf is governed by stomata and mesophyll air spaces rather than a concentrating bundle sheath. C4 plants—maize (*Zea mays*), sorghum (*Sorghum bicolor*), sugarcane (*Saccharum officinarum*), and millet—typically possess Kranz anatomy [46,47] (Figure 1), in which CO_2_ is first fixed in the mesophyll cytosol into a four-carbon acid that is decarboxylated in a chloroplast-rich bundle sheath, raising CO_2_ around Rubisco to several times the ambient rates; this suppresses photorespiration and sustains high rates under high light and temperature. A few single-cell C4 species achieve a comparable concentration of CO_2_ without a visible Kranz wreath. The classical Kranz pathway nonetheless offers several defined routes for improving the C4 engine itself, which von Caemmerer and Furbank [48] set out in detail: upregulating the carbon-metabolism enzymes phosphoenolpyruvate carboxylase, pyruvate orthophosphate dikinase, and Rubisco; increasing mesophyll carbonic anhydrase and CO_2_ porins to supply CO_2_ to the initial carboxylase; and rebalancing electron transport between the two cell types through the cytochrome b6/f complex and the partitioning of light harvesting between mesophyll and bundle-sheath chloroplasts. These targets can be pursued by transgenic modification or by selecting the natural allelic variation already present in crop populations.

The existence of these two strategies has direct consequences for nanomaterial research, and the relevant limitation changes with the environment and physiological state. First, under ambient CO_2_ and moderate light, C3 photosynthesis is frequently limited by CO_2_ supply and photorespiratory losses, so a nanomaterial that raises intercellular CO_2_, improves mesophyll conductance, or reduces photorespiration may produce a larger relative gain in C3 leaves; under high-light, high-temperature, and rising atmospheric CO_2_, however, C3 photosynthesis approaches Rubisco saturation, whereas C4 photosynthesis then becomes limited by light capture, electron transport, and sink capacity, so that a nanomaterial improving light harvesting or electron transport may be relatively more effective in C4 crops. Neither expectation is a general pathway-level rule; each applies under defined conditions and targets a specified compartment (mesophyll chloroplast for C3; bundle-sheath and mesophyll chloroplasts for C4). Second, leaf structure controls nanoparticle deposition and penetration: C4 leaves typically have smaller stomata and a lower maximal stomatal conductance, while stomatal density varies across species and a more ordered vascular network. We do not, however, treat the bundle sheath as a barrier to root-applied (xylem-borne) particles: because the bundle sheath surrounds the vascular tissue, such particles reach bundle-sheath cells first rather than having to cross them. C4 subtypes also differ: in NADP-ME crops (maize, sorghum, sugarcane) bundle-sheath chloroplasts are largely deficient in photosystem II, whereas NAD-ME and PEP-CK crops differ, so a single C4 electron-transport target cannot be assumed. Third, the two types differ in antioxidant physiology and stress responses, which determines how nanoparticles that generate reactive oxygen species (ROS) are handled [28,49]. These differences are not marginal; they can change the magnitude of a nanomaterial effect, and whether they can also change its direction is examined in Section 5 against the available direct evidence. Crassulacean acid metabolism (CAM) crops fall outside the C3–C4 scope of this review.

To make the above synthesis auditable rather than impressionistic, we first specify how the evidence base was assembled and appraised. We searched Web of Science Core Collection, Scopus, and Google Scholar, with a final search date of July 2026, using: (“nanoparticle” OR “nanofertilizer” OR “carbon dot” OR “nanomaterial”) AND (“photosynthes” OR “chlorophyll fluorescence” OR “net photosynthetic rate” OR “electron transport rate” OR “Rubisco”) AND (“C3” OR “C4” OR “rice” OR “wheat” OR “maize” OR “sorghum” OR “soybean” OR “sugarcane” OR “millet”). We included peer-reviewed research articles and reviews reporting at least one photosynthetic endpoint (net photosynthetic rate, Fv/Fm, effective quantum yield of PSII, electron transport rate, chlorophyll content, biomass, or yield) in a C3 or C4 crop under engineered nanomaterial exposure; we excluded conference abstracts, editorials, non-English records, and non-crop algae/weeds except where isolated-chloroplast or spectroscopic evidence was directly relevant to crops. We did not perform a formal meta-analysis. Instead, we (i) separated direct head-to-head C3–C4 comparisons (both types measured under matched conditions) from indirect comparisons across separate studies, and (ii) labeled each proposed mechanism in the tables as measured (by gas exchange, chlorophyll fluorescence, or biochemical assay) or as inferred by the original authors. The direct head-to-head studies are presented separately in Section 4.

## 3. Nanomaterial Entry, Translocation, and Biotransformation

Several syntheses now frame the movement of nanomaterials through plants in mechanistic rather than purely descriptive terms. The plant cell wall is a more restrictive barrier than the membranes familiar from animal-cell studies, and the precise transport mechanisms across it remain incompletely resolved [50]. Zhao and co-workers arrange nanoparticle–plant interactions around three coupled processes, translocation, transformation, and metabolic consequence, and show that during migration, particle size and surface electrical properties are the dominant determinants, with membrane interaction as a second key control; the nanoscale pores of the cell wall admit only sufficiently small particles, typically on the order of tens of nanometers or less, and transformation within the plant is driven principally by root exudates, biofilm interactions, and intracellular redox substances [51]. Xu and co-workers broaden this into a single framework, linking uptake, transport, physiology, and environment. They stress that foliar uptake is greatest when a formulation fits its entry route, the properties of the spray, and the traits of the leaf—whether penetration is cuticular or stomatal, how droplets wet and deposit, and how rough and waxy the leaf surface is [52].

How an engineered nanomaterial moves through a plant is determined above all by how it is introduced. When leaves are sprayed, particles settle on the cuticle and either penetrate it directly or pass through a stoma; once within, they travel in the apoplast, and some gain entry to the phloem, which transports them to sink tissues such as developing leaves, roots, and fruits [24]. The root route is organized differently. Particles crossing the cortex move through the apoplast or symplast until the Casparian strip deflects a portion of them, while those that enter the xylem are carried upward to the shoot in the transpiration stream [53,54]. Neither route is static. Particle size, surface charge, and coating each alter how readily particles move, and the natural organic matter in the soil solution introduces a further variable, since it can stabilize particles, dissolve them, or modify their speciation [24,55]. A wider account of the present state of plant nanobiotechnology and the questions still open can be found in [56].

An emerging consensus holds that the transformations a nanomaterial undergoes are at least as important as its pristine identity. In soil–plant systems, metal-based nanoparticles dissolve, release ions, and react with phosphates, sulfides, and organic ligands, so that the species actually taken up may be ionic, particulate, or a mixture of both [53,54]. The environmental behavior and ecotoxicity of engineered nanoparticles toward algae, plants, and fungi were systematized early in the field [57]. The dynamic transformation of nano-MoS_2_ in a soybean system, for example, empowered multiple functions on plant growth, illustrating that transformation products rather than the pristine particles mediated the observed effects [58]. The rhizosphere microbiome adds another layer of complexity: microbes transform nanoparticles and are in turn altered by them, and the resulting feedback loops make it difficult to assign causes and effects [54]. Contact with plant fluids also gives particles a biomolecular corona-a layer that defines much of their biological identity. The idea comes from nanomedicine, and work in plants has only just started [55].

For photosynthesis research, this matters because the dose that reaches the leaf or chloroplast is not the dose added to the substrate, and the chemical species actually acting on the cell is often unknown. Most papers state only the nominal concentration of the suspension applied; uptake, distribution, and speciation go unmeasured [27,44]. Cerium oxide nanoparticles added to roots, for example, largely stay in the roots and move to shoots only in small amounts, so root tissue can carry a heavy load while foliar endpoints show little response [20,59]. Small carbon dots behave in the opposite way: they move readily through the plant, reach the mesophyll and sometimes the chloroplasts, and for that reason are often chosen as photosynthetic enhancers [10,43]. As long as tissue dose is rarely measured, comparisons across materials, species, and experiments can be no more than semiquantitative.

Seed priming is a third option, and one that the nano-photosynthesis literature has largely overlooked. Dry seeds are imbibed in a nanoparticle suspension and then dried back; during imbibition particles enter through the micropyle and seed coat, are taken up by the emerging radicle, and can accompany the developing seedling [60,61]. Whether priming-derived particles reach seedling chloroplasts is unresolved, but downstream photosynthetic effects are measurable: wheat seeds primed with 10 mg/L ZnO showed higher chlorophyll a, b, and total chlorophyll, and fast chlorophyll-a fluorescence 30 days later revealed improved performance of the PSII donor-side water-splitting complex (Fv/Fo), more active reaction centers, and higher absorption, excitation-trapping, and electron-transport flux per reaction center [62]. Seed nanopriming therefore sets photosynthetic machinery in motion at the earliest developmental stage; the signaling basis is beginning to be resolved, as ZnO priming raises H_2_O_2_ as a signal molecule that enhances alpha-amylase activity and primes PSII photochemistry even under subsequent drought [63], and future studies should measure both germination and early seedling gas exchange and fluorescence after priming.

## 4. Mechanisms of Nano-Enabled Photosynthetic Regulation

Throughout this section we distinguish direct effects, in which nanoparticles interact physically or chemically with the photosynthetic apparatus itself (the pigment antenna, photosystems, electron carriers, or Calvin-cycle enzymes), from indirect effects mediated by changes in the plant physiological state (stomatal signaling, water relations, ROS homeostasis, nutrient availability, or stress priming). Direct effects are read primarily through chlorophyll fluorescence, electron transport, and enzyme assays; indirect effects are read through stomatal conductance, gas exchange, pigment content, and stress markers. Many whole-leaf responses contain both.

**Figure 2:**
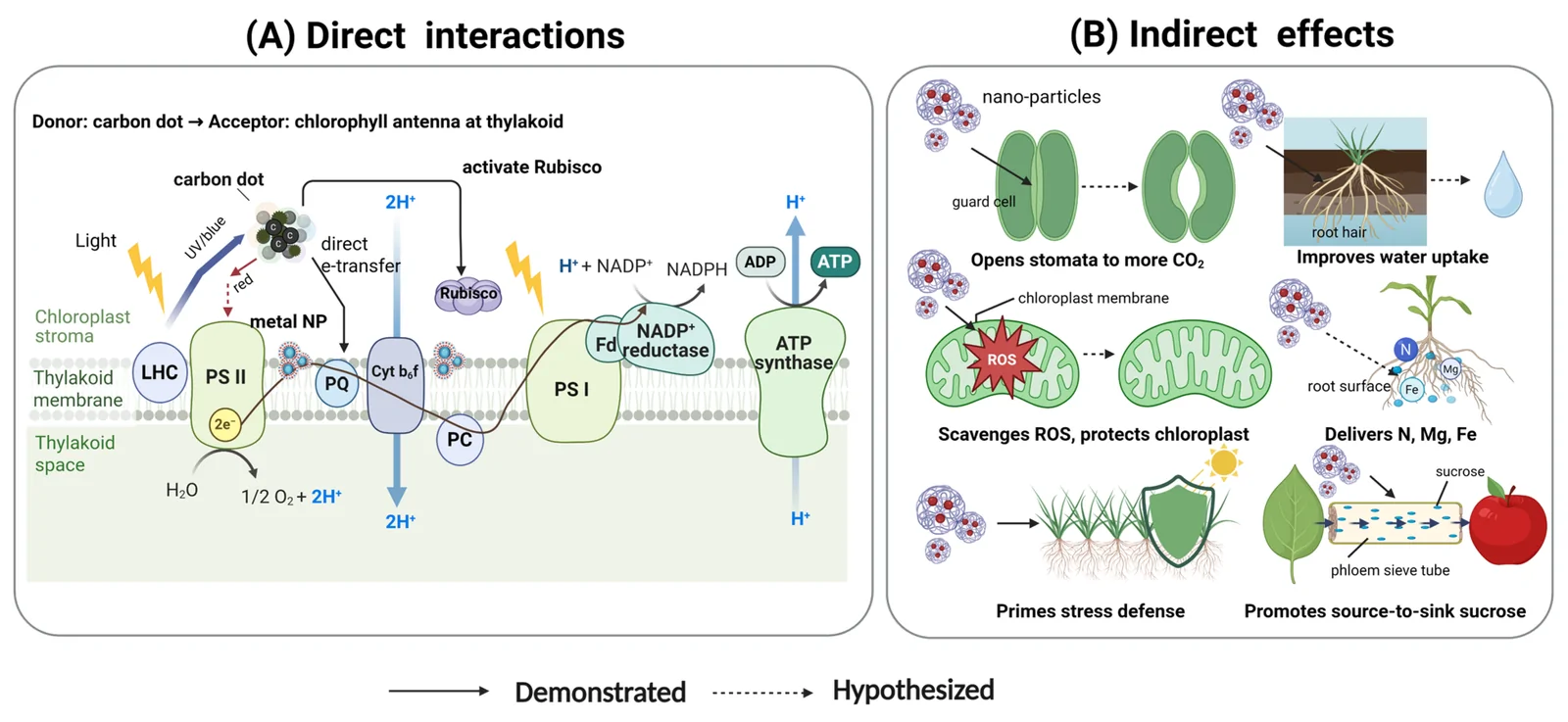
Direct and indirect mechanisms underlying nanomaterial-enhanced photosynthesis in crops. (**A**) Direct interactions at the thylakoid. Carbon dots absorb UV/blue light and down-convert it to red emission overlapping chlorophyll absorption; they can also donate electrons directly to the electron transport chain and activate or stabilize Rubisco. The canonical linear electron flow—PSII → PQ → Cytb_6_f → PC → PSI → Fd → NADP^+^ reductase—produces NADPH, while the proton gradient through ATP synthase produces ATP; metal-based nanoparticles are also shown. (**B**) Indirect effects mediated by plant physiological state: stomatal opening for CO_2_ uptake, root water uptake, ROS scavenging and chloroplast-membrane protection, the delivery of N, Mg and Fe, stress-defense priming, and source-to-sink sucrose translocation. Solid arrows denote demonstrated (measured) processes; dashed arrows denote hypothesized or incompletely established links. Created in BioRender: https://BioRender.com/1p9kxef (accessed on 26 October 2026).

Figure 2 organizes these mechanisms into two complementary panels. Panel A depicts the direct interactions that occur at or across the thylakoid membrane—the canonical linear electron chain from PSII to NADP^+^ reductase and ATP synthase, together with the three routes by which carbon dots engage this apparatus (photon down-conversion and reabsorption, direct electron donation, and Rubisco activation) and the parallel action of metal-based nanoparticles. Panel B collects the indirect routes, in which nanomaterials alter the physiological state of the plant-stomatal aperture, water relations, redox balance, nutrient supply, stress readiness, and assimilate partitioning—so that photosynthesis is supported rather than augmented at the apparatus itself.

### 4.1. Light Capture and Photon Conversion

The light reactions convert absorbed photons into chemical energy and are the first target of many nano-enabled effects (Figure 2). Metal and metal–oxide nanoparticles interact with the light reactions at several identifiable sites. The first is the pigment antenna: low doses of anatase and rutile TiO_2_ modulated photosynthetic pigments and their regulatory genes in a dose- and phase-dependent manner [13], and TiO_2_ exposure-altered growth, antioxidant response, and pigment content in fragrant rice under different light and nitrogen regimes [15]. The second site is the photosystem itself, and it is the most direct. Hong and co-workers demonstrated this as early as the spinach experiments, in which nano-TiO_2_ increased the photochemical activity of chloroplasts, oxygen evolution, and the functioning of photosystem II [64]. A range of other metallic nanoparticles has since been shown to change chlorophyll fluorescence and electron transport, and occasionally the structure and functioning of the entire photosynthetic apparatus [7,65]. These findings can be interpreted at the membrane level. Metals bind to thylakoid membranes and alter the charge environment surrounding PSII reaction centers; a metal that is redox-active may also enter into electron transfer or generate ROS. Which of these pathways dominates seems to depend on dose and particle shape. The tomato experiments make the role of shape and coating plain. Two ZnO particles, both capped with oleylamine and applied at similar exposure, produced opposite results—the rods depressed PSII photochemistry while the irregular particles enhanced it [66]—so morphology and surface chemistry alone can reverse the sign of a response. Dose then determines how far the response extends: in Brassica juncea, foliar CuO at 8 mg/L gave the best results for chlorophyll, net photosynthesis, and antioxidant enzymes [67], whereas heavier metal loads compromise thylakoid integrity and the antioxidant network that shields the photosystems [65]. Whether C3 and C4 plants differ in these metal responses is examined in Section 5: the separate mesophyll and bundle-sheath compartments, and the PSII deficiency of NADP-ME bundle-sheath chloroplasts, mean that a metal acting on PSII is not expected to affect the two types identically. Titanium dioxide has been comprehensively reviewed with respect to synthesis, agricultural applications, and toxicity [68], and a further role for TiO_2_ in reprogramming metabolism to mitigate UV-B stress is discussed in Section 4.3 [69].

Carbon dots absorb UV and short-wavelength visible light and emit at longer wavelengths that overlap the chlorophyll Soret and Q bands; this is down-conversion (Stokes-shift emission), and anti-Stokes up-conversion has not been demonstrated for these particles in planta. We distinguish three quantities that are often conflated as “quantum yield”: (i) the photoluminescence quantum yield of the carbon dots themselves (measured in solution); (ii) the photochemical yield of photosystem II (Fv/Fm and PhiPSII, measured by chlorophyll fluorescence in intact leaves); and (iii) the net carbon-assimilation yield (measured by gas exchange). These three quantities are related but distinct, and each is reported separately. Four routes are kept separate: (a) the down-conversion and reabsorption of emitted light, supported by fluorescence spectra in vitro and, in some cases, in planta; (b) Forster resonance energy transfer from carbon dots to chlorophyll, which is best supported in vitro and difficult to demonstrate unambiguously in intact leaves; (c) direct electron transfer, supported in planta by the apple study in which N-doped carbon dots donated electrons to plastoquinone-9 [70]; and (d) indirect nutritional or hormone-like signaling. We do not infer one route from evidence obtained for another. An early in planta study by Li and colleagues found that a moderate photoluminescence quantum yield (46.42%) was optimal, as both lower (15.13%) and higher (90.59%) yields were unfavorable, with the former competing with chloroplasts for light and the latter exceeding the chloroplast saturation point; the moderate-yield carbon dots accelerated electron transport, raised Rubisco activity, and increased biomass in rice while acting only as light converters without transferring energy to isolated chloroplasts [10]. Systematic reviews caution that reported “up-conversion” and energy-transfer efficiencies are often measured in solution and do not always survive in intact leaves [11]; dual-emissive carbon dots coated on isolated chloroplasts raised ATP 2.8-fold and, in intact leaves, raised the electron transport rate by up to 25% [71]. This down-conversion behavior has been exploited in biohybrid systems in which biomass-derived carbon dots closed the loop between light harvesting and carbon fixation, enhancing photosynthesis in a continuous manner [43]. High-photon-harvesting nanophotofertilizers have been designed to regulate plant growth by improving light utilization across the visible spectrum [72]. Nitrogen doping enhanced the light-conversion and electron-supply functions of carbon dots: foliar application of N-doped carbon dots increased the net photosynthetic rate by 21.51% and yield by 24.50% in maize [73]. Magnesium–nitrogen co-doped carbon dots entered chloroplasts and acted as energy-transfer agents, increasing photosynthetic activity, electron transport rate, and Rubisco activity in rice [74]. The broader nanobionics agenda, which aims to embed nanomaterials into living plant or algal systems to augment their intrinsic photosynthetic capacity, has been reviewed in detail for both crops and microalgae [75]. This agenda originated with the demonstration that single-walled carbon nanotubes embedded in isolated spinach chloroplasts enhanced photosynthetic electron transport rates more than threefold, introducing the concept of plant nanobionics [76]. This agenda has advanced from concept to proof in planta: soil-applied, glucose-functionalized carbon dots improved photosynthesis and increased grain yield by 18% in dwarf bread wheat [77]. A critical review of the performance of carbon dots, however, cautioned that gains are often modest, that many studies lack appropriate controls for the ionic or organic precursors, and that the assigned mechanisms are not yet settled [11]. Whether carbon dots enter chloroplasts at sufficient concentrations to act as energy-transfer agents in vivo, rather than merely as micronutrients or hormone-like signals, is an active question [11,42,78]. A recent study in apple has begun to close this gap by showing that nitrogen-doped carbon dots can donate electrons to plastoquinone-9 in the photosynthetic electron transport chain, providing direct mechanistic evidence for carbon-dot participation in electron transfer [70].

The magnitude of any photon-conversion benefit also depends on the light environment in which a plant is grown and measured. Carbon dots absorb UV and short-wavelength blue light, the part of the spectrum that instrument LEDs and many controlled-environment chambers provide only weakly. A sunlit canopy and a growth cabinet therefore supply quite different numbers of photons for down-conversion. Whether a measured gain comes from photon conversion or from a nutritional or signaling effect can be traced only in studies that report the incident UV and blue flux, the carbon-dot loading inside leaves, and the spectral overlap between dot emission and chlorophyll absorption. When the light source contains no UV, any rise in assimilation can then be attributed to those latter pathways. This dependence on the ambient spectrum goes some way toward explaining why the same dots produce apparently different gains in different studies.

Other carbon nanomaterials reach the photosynthetic apparatus by quite different routes. Single-walled carbon nanotubes embedded in isolated spinach chloroplasts raised photosynthetic electron transport more than threefold in the first plant-nanobionics experiment of this kind [76]. The photosynthetic effects of graphene oxide (GO) have been measured directly in wheat seedlings. GO changed net photosynthetic rate, stomatal conductance, Fv/Fm, the effective PSII quantum yield, electron transport rate, and Rubisco content, all in a dose-dependent fashion. Above roughly 20 mg/L it acted on its own, while below that it strongly worsened the photosynthetic inhibition caused by cadmium [79]. Fullerenol, hydroxylated C60, shows how the same carbon family can give opposite results depending on formulation and context. In cucumber, C60 fullerenols changed the leaf metabolite and protein profile, yet when copper was present, they increased copper uptake and made copper toxicity worse rather than relieving it. The result is a warning that protein-level changes alone do not indicate a net benefit [80]. The clearest beneficial effects of fullerenol occur under micronutrient deficiency: in maize (C4), fullerenol alleviated iron-deficiency chlorosis by mobilizing root apoplastic iron and raising leaf active iron, while leaving iron-sufficient plants unchanged, i.e., the effect is recovery from deficiency rather than stimulation under non-limiting nutrition [81]. In rice, reduced graphene oxide, multi-walled carbon nanotubes, and C60 at 10–250 mg/L reshaped the fungal endophyte community even at the lowest concentration, with the largest changes at 250 mg/L, showing that plant-associated microbial communities can respond before the plant itself shows any visible change [82].

### 4.2. Carbon Assimilation and the Calvin–Benson Cycle

The dark reactions convert CO_2_ into sugars and are the ultimate determinant of biomass gain. Nanomaterials can influence carbon assimilation through several distinguishable routes, each resting on a different evidence base. The first acts on inorganic-carbon supply. Particles that dissolve in the rhizosphere can enter carbonate equilibria, and metal-based nanoparticles such as ZnO stimulate the release and microbial decomposition of dissolved organic carbon (1.8 to 2.4 fold in a legume rhizosphere), together with the activity of carbon- and nutrient-cycling enzymes, reshaping the carbon and nutrient environment in which assimilation proceeds; the latter is more clearly documented [83]. A more direct strategy recruits carbonic-anhydrase-mimetic nanozymes: Zn-based frameworks such as ZIF-8 both adsorb CO_2_ and catalyze its hydration to bicarbonate, raising CO_2_ around Rubisco and upregulating the enzyme, a mechanism first established by the structural and functional mimicry of the zinc carbonic-anhydrase center in ZIF-8 [84], and demonstrated in microalgae and in vitro [85]. The second route acts on Rubisco and the cycle enzymes directly: Mg-N co-doped carbon dots in rice [74] and graphene oxide in wheat [79] each increased or protected Rubisco abundance or activity, whereas silver nanoparticles provide the converse demonstration by inhibiting the particle-sensitive, NADP-dependent glyceraldehyde-3-phosphate dehydrogenase in *Spirodela polyrhiza* [86]. Proteomic studies document the same enzyme-level specificity more broadly: ZnO altered chloroplast proteins of electron transport and ATP production in bean [87], and anatase TiO2 induced a covalent Rubisco large-subunit dimer together with the loss of cytochrome f and chlorophyll a/b-binding proteins in tobacco [88]. The third route concerns regeneration of ribulose-1,5-bisphosphate, which is set by the ATP and NADPH supplied by the light reactions (Section 4.1). Because direct in-crop biochemical assays remain fewer than whole-leaf gas-exchange observations, each route is most usefully read against the system in which it was established-intact plant, isolated chloroplast, or in vitro. The carbonic-anhydrase route, demonstrated in microalgae and in vitro, is a particularly promising candidate to carry into crop leaves, and paired experiments that measure both CO_2_ hydration and assimilation in the same plants would be a valuable direction for future work.

Photosynthetic stimulation at the whole-leaf level has been documented for several nanoparticles, and the mechanisms can now be traced from the leaf to the sink. Foliar nano-carbon dots raised rice grain yield under nitrogen deficiency by strengthening leaf photosynthesis and the movement of stem assimilates. The response in rice did not act on yield in isolation but could be followed from leaf to grain, as a stronger source supplied a larger sink [89]. In tomato, the same dots extended the photosynthetic life of leaves, and growth and yield followed [90]. Lettuce offers yet another pathway. When zinc was paired with carbon dots, the nanofertilizer acted on leaves and roots together and raised both yield and quality [91]. The common thread across all three crops is that yield never improves by itself: net photosynthetic rate, PSII quantum yield, or chlorophyll always changes first. Iron-based particles fit into this pattern as stress treatments, not as growth promoters. In peanut grown under salt stress, iron oxide treatment brought about a joint recovery of photosynthesis, osmoregulation, and antioxidant activity [22], whereas soybean receiving foliar ferroferric oxide displayed closer source–sink coupling and improved growth [21]. In tea plants, ZnO enhanced photosynthesis and shoot development and at the same time reshaped the phyllosphere microbiome, thereby connecting leaf physiology to the microbial community on the leaf surface [18]. These reports are encouraging but heterogeneous: stimulation was quantified with different techniques (gas exchange, chlorophyll fluorescence, pigment content, biomass), at different times after application, and rarely with a full carbon balance, so the magnitude of the dark-reaction response remains uncertain until measurements are harmonized [11,27]. At the whole-plant level, the broader growth and physiological responses of plants to metal and metal–oxide nanoparticles are reviewed by Rizwan and colleagues [92], and representative whole-leaf outcomes are compiled in Table 1.

The studies compiled in Table 1 show a consistent pattern: photosynthetic enhancements are reported mainly at low doses, often with stress-priming or nutritional mechanisms, whereas inhibitory outcomes cluster at higher doses. This correspondence between dose and mechanism argues for reading enhancements together with the physiological context of exposure. At present, the majority of single-species evidence derives from C3 crops, while direct measurements in C4 crops are still scarce. For this reason, the matched comparisons are gathered in a separate comparative section below, and the principles specific to C4 crops in Section 5 follow the same reasoning.

### 4.3. Stomatal Behavior, Water Relations, and ROS Homeostasis

Stomatal conductance is the point at which CO_2_ enters and water exits, and it is a frequent site of nanomaterial action. Particles alter stomatal opening in one of two ways—by acting directly on guard-cell signaling, or by changing the plant’s water status and its handling of abscisic acid. In lettuce, TiO_2_ nanoparticles enabled cells to withstand UV-B stress via pigments and antioxidant enzymes rather than through damage [69]. In wheat, an appropriately balanced dose of nano-macronutrients improved growth, productivity, and quality by adjusting antioxidant activity [95]. Drought damage is likewise reported to diminish when water relations, osmoregulation, and photosynthesis get better [35,96,97], and the same is true under salt stress [16,22]. Chitosan–selenium sprays aided bitter melon under salt stress, increasing antioxidant enzymes, proline, and leaf water while lowering MDA and H_2_O_2_ [98]. In rice, ZnO alleviated arsenic toxicity: growth and photosynthesis improved, and shoot arsenic declined by as much as 40.7% [99]. Black cumin subjected to drought together with chromium did better with silicon and titanium particles, with effects extending to photosynthetic membranes and lipid metabolism [100]. In chromium-exposed rice, ZnO limited chromium uptake, repaired chloroplasts, and lessened oxidative stress [101]. The same pattern recurs: a brief, modest burst of ROS from nanoparticles activates the antioxidant system, leaving the plant better prepared for subsequent stress. Such “stress training” has been documented in rice using nanobiostimulants [94] and is consistent with the broader evidence that nanomaterials aid stress resistance [36,37,102].

It is tempting to interpret these observations as hormesis, but caution is warranted. TiO_2_ nanoparticles produce biphasic responses, with low doses stimulating and high doses inhibiting—the pattern that hormesis describes [14]. Cerium oxide nanoparticles act in much the same manner, scavenging ROS at low concentrations but behaving as oxidants at high ones [20,59]. The unresolved question is whether the stimulatory phase genuinely strengthens the photosynthetic machinery or merely reflects a stress response that happens to be beneficial over the short term. The two readings can be separated only by measuring the complete dose–response curve, its descending branch included, and by following plants past a single harvest [29,45].

These stomatal and oxidative responses vary in both direction and magnitude, depending on concentration, particle morphology, dose, and timing rather than on material identity alone. A factorial exposure of lettuce to silver nanoparticles differing in size (60, 75, and 100 nm), surface coating, and concentration (1–15 mg/L) showed that root accumulation followed size and concentration but not coating, whereas the effects on transpiration and stomatal conductance emerged only at the higher concentrations, indicating that a stomatal response is expressed only after a tissue threshold is crossed [103]. Copper oxide shows a similar concentration-dependence. In lettuce and cabbage, plants tolerated low foliar doses, but 250 mg per plant left copper-rich necrotic lesions around deformed stomata, lowered both net photosynthesis and leaf water content, and the foliar transfer rate climbed from 7.8 to 242 µg per day as exposure went on [104]. Shape and surface chemistry enter the picture alongside dose. Spherical biogenic copper nanoparticles (29–79 nm) at 100 µg/mL adjusted stomatal immunity and kept reactive oxygen in balance rather than overwhelming it; the same metal at higher doses, with less controlled morphologies, instead produces oxidative damage [105]. The developmental stage at which the material is applied matters as well. In wheat, three low doses of iron–porphyrin hybrids given during germination and tillering changed pigment content, lipid peroxidation, flavonoids, and the phenolic profile in stage-specific ways, and gas exchange improved only during tillering. The same formulation can thus be neutral or beneficial depending on when it is applied [106]. Taken together, these studies suggest that the variables do distinct jobs. Concentration and dose decide whether a particle primes or causes toxicity; shape and surface chemistry decide how it engages guard-cell and thylakoid membranes; and application period and rate decide the metabolic state it meets on arrival. When all of these are reported, a stomatal effect can be interpreted rather than merely recorded.

### 4.4. Source–Sink Coordination and Yield Formation

Stronger photosynthesis is of little agronomic value unless the extra assimilates actually reach the organs that are harvested. A growing number of studies show that nanomaterials can improve this source–sink coordination. When carbon dots were applied to rice under nitrogen deficiency, they enhanced both leaf photosynthesis and the movement of assimilates from the stem, linking the source and sink phases [89]. Ferroferric oxide nanomaterials enhanced source–sink communication in soybean, with yield gains beyond what photosynthesis alone would predict [21]. A two-year field study in rice showed that a single foliar application of ZnO nanoparticles increased grain yield by 1.4–4.6%, enhanced the net photosynthetic rate after heading, improved grain quality, and raised zinc content by up to 85% in brown rice and 103% in polished rice [107]. Nanofertilizers in general have been proposed to act through multiple dimensions, including nutrient supply, hormone-like signaling, and altered partitioning, with effects on both vegetative and reproductive organs [30,34,108]. The current state and future perspectives of nanofertilizers for precision and sustainable agriculture have been reviewed [109]. The multidimensional regulation mechanism of nanofertilizers on vegetable quality has been summarized systematically [34], and the molecular mechanisms of plant productivity enhancement by nanofertilizers have been reviewed at the gene-expression level [110]. Yet the number of studies that simultaneously measure photosynthesis, assimilate partitioning, and final yield remains small, and even fewer trace the fate of the added nanomaterial in the harvested product [111]. A simultaneous increase in leaf photosynthesis and yield is therefore a consistent association, but it supports a source-to-sink causal chain only when partitioning, stem-to-grain transport, and the underlying enzymes or genes are also measured, ideally with temporal or perturbation evidence. The interactions of nanomaterials with secondary-metabolite accumulation, photosynthesis, and nitrogen fixation are equally complex, and their coordinated regulation is only beginning to be mapped [112]. In the absence of such information, claims of enhanced yield rest on a causal chain whose links are incomplete [33,108]. Table 2 summarizes representative yield and quality outcomes.

Table 2 indicates that yield and quality benefits are most consistently reported when a nano-fertilizer provides a nutrient that is limiting or corrects a deficiency, and that quality traits—grain protein, micronutrient content, or antioxidant accumulation—frequently improve together with yield. As in Table 1, gains depend on dose, timing, and application route; they show that a well-designed nano-fertilizer can match nutrient supply to crop demand, not multiply yields universally.

**Figure 3:**
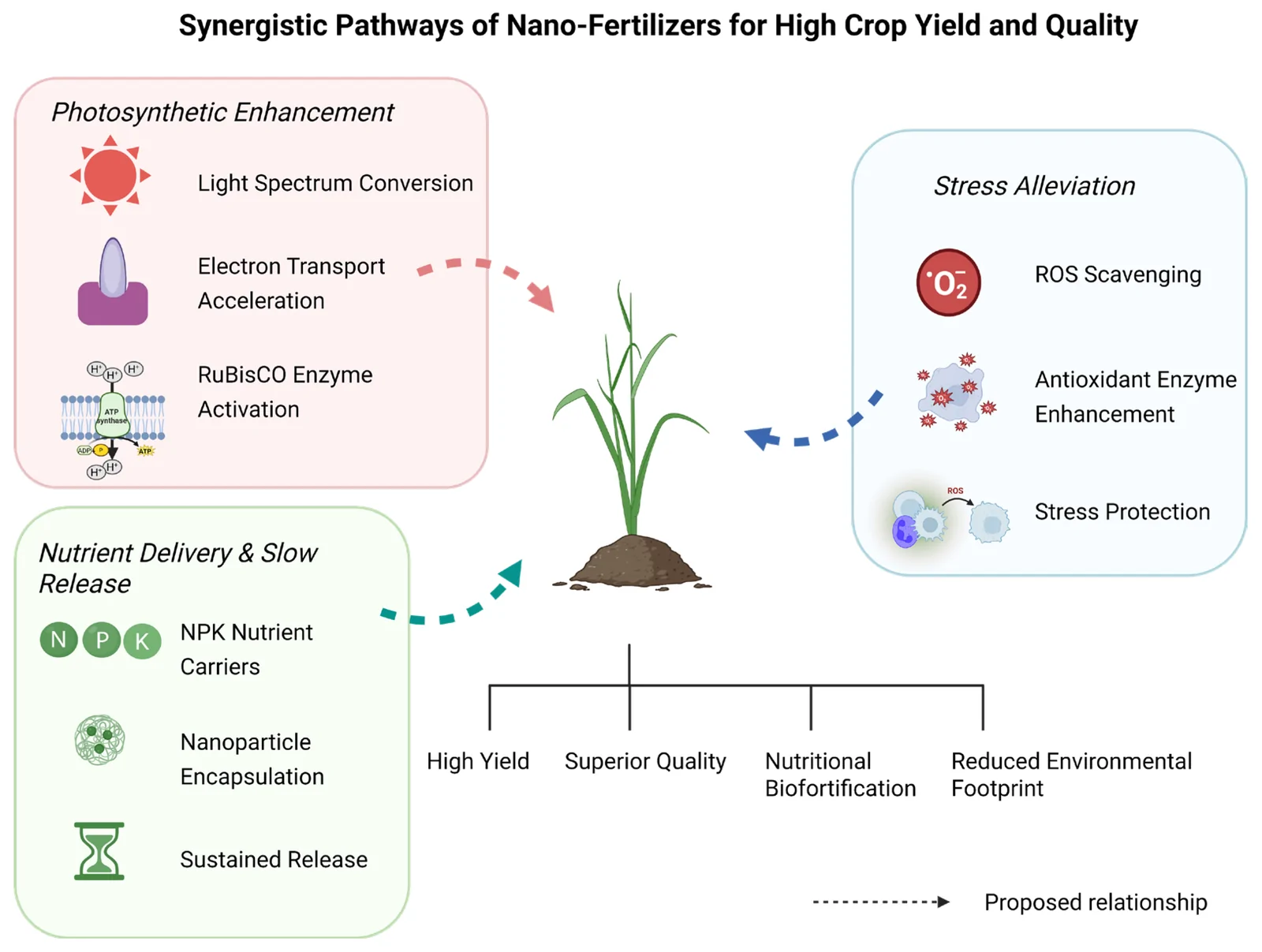
Synergistic pathways of nano-fertilizers for high crop yield and quality. Photosynthetic enhancement, nutrient delivery and slow release, and stress alleviation may lead to higher yield, better quality, nutritional biofortification, and a smaller environmental footprint. Dashed arrows mark proposed rather than established links, as defined in the legend. Created in BioRender: https://BioRender.com/2ori6nh (accessed on 26 October 2026).

Alongside single-species work, a small but increasing number of experiments directly compare C3 and C4 crops exposed to the same nanomaterial. Because both types are measured in the same chamber, at the same dose, and for the same duration, the C3/C4 contrast is not confounded by growth conditions, and these direct head-to-head studies are summarized in Table 3. No simple tolerance ranking emerges from these studies. Within each study the two crop types respond in the same direction, both enhanced by light-converting carbon quantum dots and both inhibited by the metal–oxide and arsenite treatments, while the magnitude of the response differs between them. In the La_2_O_3_ study maize was more strongly inhibited than soybean despite lower whole-plant (and root) lanthanum uptake, and under nanoparticle stress C4 crops tended to benefit more from elevated CO_2_, indicating that which type responds more strongly follows the photosynthetic step targeted rather than photosynthetic type alone. An earlier head-to-head study using carbon quantum dots derived from empty-fruit-bunch biochar and applied to rice and corn increased CO_2_ assimilation and stomatal conductance in both crops, directly comparing the C3 and C4 response to foliar carbon dots [113]. Each mechanism in Table 3 is labeled as measured (by gas exchange, chlorophyll fluorescence, biochemical assay, or metabolite profiling) or as inferred by the original authors. The synergistic pathways by which nanofertilizers promote yield and quality are illus-trated in Figure 3.

Nanomaterials interact with the photosynthetic apparatus at identifiable components rather than acting on a single black-box process. At the light reactions, metal and metal–oxide nanoparticles can associate with thylakoid membranes, modify the charge environment around PSII reaction centers, and, for redox-active metals, participate in electron transfer or ROS chemistry; the balance of these actions is set by dose and particle shape, so the same material can enhance or inhibit PSII depending on morphology and surface chemistry, as when rod- and irregularly shaped ZnO exert opposite effects in tomato. At carbon assimilation, nanomaterials act through three distinguishable routes—inorganic-carbon supply, Rubisco and cycle enzymes, and RuBP regeneration—where carbonic-anhydrase–mimetic Zn frameworks raise CO_2_ around Rubisco while silver nanoparticles provide the converse by inhibiting NADP-dependent GAPDH. Across photosynthetic types, the mesophyll chloroplast of C3 plants and the dual mesophyll/bundle-sheath compartments of C4 plants, together with the PSII deficiency of NADP-ME bundle-sheath chloroplasts, imply that a particle acting on PSII need not affect the two types identically.

## 5. Differential Responses of C3 and C4 Crops: Toward Design Principles

The central argument of this review is that C3 and C4 crops may respond differently to the same nanomaterial, and that photosynthetic type provides a principled basis for interpreting those responses. Direct comparative evidence is limited, but it shows a consistent pattern: within each head-to-head study the two crop types respond in the same direction, both enhanced by light-converting carbon quantum dots and both inhibited by the metal–oxide and arsenite treatments, while the magnitude of the response, the degree to which elevated CO_2_ mitigates it, and the photosynthetic step most affected differ between them. Direct comparative evidence is summarized in Table 3. In the most direct comparison to date, soybean (C3) and maize (C4) exposed to La_2_O_3_ nanoparticles showed declines in net photosynthetic rate of 8.77% and 55.52%, respectively, with PEPC and Rubisco suppressed only in maize [114]. Elevated CO_2_ differentially mitigated indium oxide nanoparticle-induced oxidative stress in young and old leaves of C3 and C4 crops, showing that atmospheric CO_2_ interacts with photosynthetic type to determine sensitivity [49,115]. Reviews of nanomaterials and photosynthesis in gramineous plants have likewise noted that responses differ among C3 and C4 grasses [117].

Three mechanistic explanations account for these differences. First, the CO_2_-concentrating mechanism of C4 plants makes their photosynthesis less sensitive to CO_2_ supply but more dependent on light and electron transport; nanomaterials that primarily increase CO_2_ availability or suppress photorespiration should therefore benefit C3 plants more, whereas nanomaterials that improve light harvesting or electron transport should benefit C4 plants more. Second, the antioxidant systems of C3 and C4 plants differ in capacity and inducible responsiveness, so that ROS-generating nanoparticles may be detoxified differently in the two types [49]. Third, the spatial organization of the Kranz pathway places the relevant target steps in different compartments rather than placing a barrier in front of them. In C4 leaves, the bundle sheath surrounds the vascular tissue, so particles delivered through the xylem encounter the sheath alongside or before the mesophyll; which compartment is reached first is therefore set by the delivery route and not by the sheath acting as a barrier to root-applied particles. Because the bundle-sheath chloroplasts of the common NADP–malic–enzyme C4 subtypes are largely deficient in photosystem II, a nanomaterial that acts on PSII or the oxygen-evolving complex targets different steps in different C4 subtypes [28,116].

These differences yield practical design rules. A nanomaterial meant to enhance photosynthesis in C3 crops ought to favor mechanisms that raise CO_2_ concentration at Rubisco, diminish photorespiration, or improve mesophyll conductance; carbon dots that improve light utilization and stress priming are particularly suitable candidates [11,12]. For C4 crops, the corresponding priorities are electron transport, sink strength, and stress tolerance. These C4 priorities are grounded in the C3 evidence base, ZnO in rice [16], Fe_3_O_4_ in soybean [21], and balanced nano-macronutrients in wheat [95], and are extended to C4 by the N-doped carbon-dot study in maize, which raised net photosynthesis by 21.5% and yield by 24.5% [73]. The C4 priorities target the bundle-sheath and mesophyll chloroplasts and are ready for evaluation in paired C3/C4 trials. Experimental studies of foliar uptake across leaf anatomies that differ from one another show that size and coating chemistry determine whether entry is stomatal or cuticular and how particles move from leaf to rhizosphere [118]; that the composition of protein coatings directs particles toward stomata and trichomes [119]; and that charge and size govern delivery into guard cells, the extracellular space, and chloroplasts, with size cut-offs that differ between cotton (a dicot) and maize (a monocot) [120]. These mechanisms are consistent with the carbon-dot evidence base, which documents improved light utilization and stress priming across C3 crops [11,12]; paired C3/C4 trials that quantify CO_2_ at Rubisco, photorespiration, and mesophyll conductance would now allow these mechanistic expectations to be tested directly. These principles provide a practical starting point for such work; direct C3–C4 comparisons remain scarce and within-type variation may rival between-type variation [28,117]. Future experiments should include paired C3 and C4 species under identical conditions as a routine element of nano-photosynthesis research.

## 6. Dose–Response Framework, Methodological Challenges, and Field Extrapolation

### 6.1. Dose–Response Relationships and the Conceptual Photosynthetic Dose–Response Framework

The single most important determinant of whether a nanomaterial helps or harms photosynthesis is dose. Across the literature, low doses of many nanoparticles stimulate growth and photosynthesis, while high doses inhibit them, a pattern consistent with hormesis [14,29]. The hormetic dose–response has been established as a general biological principle with major implications for environmental risk assessment [121]. The stimulatory phase has been documented for TiO_2_ [13,14], ZnO [17], CeO_2_ [19,20,25], carbon dots [10,11], and nanofertilizers in general [30,33]. The evidence for inhibition is no less strong: nanoparticles delay seed germination and root growth [23], mesoporous carbon particles turn out to be toxic to rice [93], and nano pollution places a broader burden on farmland [26].

We accordingly put forward a conceptual photosynthetic dose–response framework comprising four operational zones, each defined by measured endpoints rather than by visible appearance. Zone 1: No Detectable Response. Net photosynthesis, Fv/Fm, chlorophyll content, and biomass do not differ significantly from controls within the study’s sampling. Zone 2, Adaptive Signaling/Stress Priming. ROS is generated only briefly and antioxidant enzyme activity increases, but no photosynthetic endpoint declines. Zone 3: Physiological Stress with Yield Maintained. Photosynthetic endpoints fall significantly, although biomass and yield are not yet affected. Zone 4: Adverse Effects. Photosynthetic endpoints decline significantly, accompanied by visible injury or measurable loss of yield. Not every material, however, moves through all four zones. Null responses and monotonic dose–responses occur frequently and should simply be reported rather than squeezed into the four-zone pattern. The multi-dose ZnO study in submerged rice illustrates the point [122]. In hydroponic plants, ZnO at up to 10 mg/L enhanced growth and pigments, antioxidant enzymes increased or held steady at every dose, and 25 and 50 mg/L were toxic to growth. The lesson is that a single positive or negative result, on its own, reveals little. The dose must be located on the full curve; and when risk and benefit are weighed, the width of the enhancement window matters most. This framework concerns photosynthetic dose–responses, not food safety. The risks of chronic exposure and of accumulation in edible tissues remain uncertain [44,111], so agronomic efficacy and human exposure ought to be assessed separately.

Illustrative thresholds are material- and species-specific: in rice, ZnO enhanced chlorophyll and growth at low doses and inhibited at high doses [122]; in wheat, graphene oxide alone became negative above ~20 mg/L and amplified cadmium at only 5 mg/L [79]; in rice, MWCNT, C60, and reduced graphene oxide altered the endophyte community at 10 mg/L and most strongly at 250 mg/L [82]; mesoporous carbon strongly inhibited rice root and shoot length at 150 mg/L [93]. These figures merely indicate the zone boundaries within those particular studies; they are not cut-offs that can be applied elsewhere.

The framework also reveals the limits of current knowledge. Most published studies examine only two to four doses, frequently capturing neither the ascending nor the descending branch of the response curve, so the enhancement window cannot be delineated [27]. The role of exposure duration is even less understood: short-term stimulation may reverse after chronic exposure, and the long-term fate of nanoparticles in edible tissues raises food-safety questions that have only begun to be addressed [44,111]. Finally, the framework must be extended from single materials to realistic mixtures, because agricultural soils contain multiple nanoparticles, ions, and natural colloids whose combined effects cannot be inferred from single-material experiments [44,45].

### 6.2. Methodological Challenges and Field Extrapolation Risks

Several methodological problems make nano-photosynthesis studies hard to compare. Doses are reported on different bases—per mass of soil, per volume of spray, or per plant—and are seldom converted into tissue doses [27]. Characterization is often partial as well: hydrodynamic size, zeta potential, dissolution rate, and speciation in the exposure medium are missing, even though each of these shapes the biological outcome [24,55,123]. Controls are also often incomplete. A number of studies leave out the ionic precursor or the ligand, meaning that effects attributed to nanoparticles may in fact arise from dissolved ions or organic coatings [11,29]. Nor are the photosynthetic measurements any more uniform: gas exchange, chlorophyll fluorescence, pigment extraction, and biomass are treated as though they were interchangeable, often at a single time point [7,65].

The leap from controlled environments to the field is still more problematic. Field soils are difficult to reproduce indoors. Their light, temperature, water, nutrients, and microbes all vary together, and the processes that determine the fate of nanoparticles in real soil cannot readily be reconstructed in a laboratory or greenhouse [53,54]. The broader argument for agricultural nanotechnology and its role in the agri-tech revolution is presented in [124]. Genuine field trials of nanofertilizers are still uncommon, and those that are conducted often forgo replication, randomization, or long-term monitoring [33,108]. Earthworms and other soil organisms may also be harmed, and this risk must be assessed before such products come into wide use [44]. Then there is the matter of rising CO_2_. It alters the initial physiology of both C3 and C4 crops and may change how nanomaterials respond to dose, so studies that combine elevated CO_2_ with nanomaterials—rice treated with biochar and CeO_2_, for example [125]—are needed if the results are to remain valid in coming decades.

Table 1, Table 2 and Table 3 arrange studies according to what was actually measured: gas exchange, chlorophyll fluorescence, pigment content, biomass, or yield. We also recorded whether each study controlled for the ionic form, the bulk material, the coating or ligand, or matched nutrients. Where particles were supplied to deficient or stressed plants—carbon dots under nitrogen shortage [89], FeO under salt stress [22]—we regarded the gain as recovery rather than stimulation under favorable conditions. In the same way, source–sink links in Section 4.4 count as concurrent changes unless a single study measured photosynthesis, assimilate partitioning, and yield together. Few do.

A small number of reviews have started to catalog these gaps. The field would benefit from straightforward reporting rules, trials conducted across laboratories, and open data. Until these are in place, quantitative claims should be considered alongside the evidence that supports them, and meta-analysis should be attempted only when the inclusion rules specify dose, characterization, and design [29,45].

## 7. Controversies and Open Questions

The nano-photosynthesis literature is not unanimous on every point, and to pretend otherwise serves no purpose. Consider carbon dots first. Their photon-conversion account is cited widely [10,43], yet energy transfer within chloroplasts has never been demonstrated directly by spectroscopy. The dots might instead act through nutrient-like signaling, antioxidant activity, or microbes, and none of these interpretations has been settled [11,42]. Metal-based particles present a different difficulty: when an effect occurs, it is unclear whether the particle itself does the work, the ions it releases do, or both do, and the division appears to shift from one material and exposure to another [29,53,54]. The C3–C4 comparison is equally unresolved. Photosynthetic type seems a plausible factor shaping the response [28,49], but matched studies are so few that variation within a type could be as great as variation between types [117]. Finally, the agronomic payoff remains uncertain. Many papers report short-term physiological gains and go no further; the handful that measure yield generally find small effects that fail to hold across sites or seasons [33,34,108,126]. Even nanofertilizers—the most commercially advanced of nano-enabled inputs—have not resolved whether they offer real advantages over existing products when deployed at scale [127].

Open questions arise directly from these disputes. What is the smallest effective dose for each nanomaterial, and how broad is its enhancement window under field conditions? How do nanoparticles behave in the phyllosphere and rhizosphere over an entire growing season, and what are the transformation products that actually reach the photosynthetic tissues [54]? Can carbon dots be engineered to deliver photons, nutrients, and stress signals simultaneously without accumulating in edible parts [12,42]? How do nanofertilizers interact with conventional fertilization regimes, and can they reduce nutrient losses while maintaining yield [34,108,128]? Can seed nanopriming deliver nanomaterials at the earliest developmental stage to improve germination, stress tolerance, and subsequent photosynthetic performance [60,61]? Answering these questions will require the methodological reforms outlined in Section 5 and Section 6, and, in particular, the routine inclusion of paired C3 and C4 species, full dose–response designs, harmonized photosynthetic measurements, and long-term fate studies.

## 8. Conclusions

This review has reorganized the evidence on nano-engineered photosynthetic regulation around three principles. First, the C3–C4 distinction should be treated as a candidate design variable rather than an established determinant: C3 crops generally lack Kranz anatomy and suffer photorespiratory losses, whereas C4 crops typically possess Kranz anatomy and a CO_2_-concentrating mechanism, but direct evidence that this difference systematically controls the sign of a nanomaterial response is still thin [28,49]. Second, the dose–response continuum should be interpreted within a conceptual photosynthetic dose–response framework that distinguishes a no-response zone, an adaptive-signaling zone, a physiological-stress zone, and a harmful zone, because the same nanomaterial can occupy all four zones depending on dose and context [14,29]. Third, progress depends less on new materials than on better experiments: standardized characterization, harmonized photosynthetic endpoints, paired C3–C4 designs, adequate controls, and long-term fate and yield measurements [27,44,45]. The wider program of engineering crop photosynthesis to raise yield potential, at the level of both source and sink, has been examined at length [129,130].

**Figure 4:**
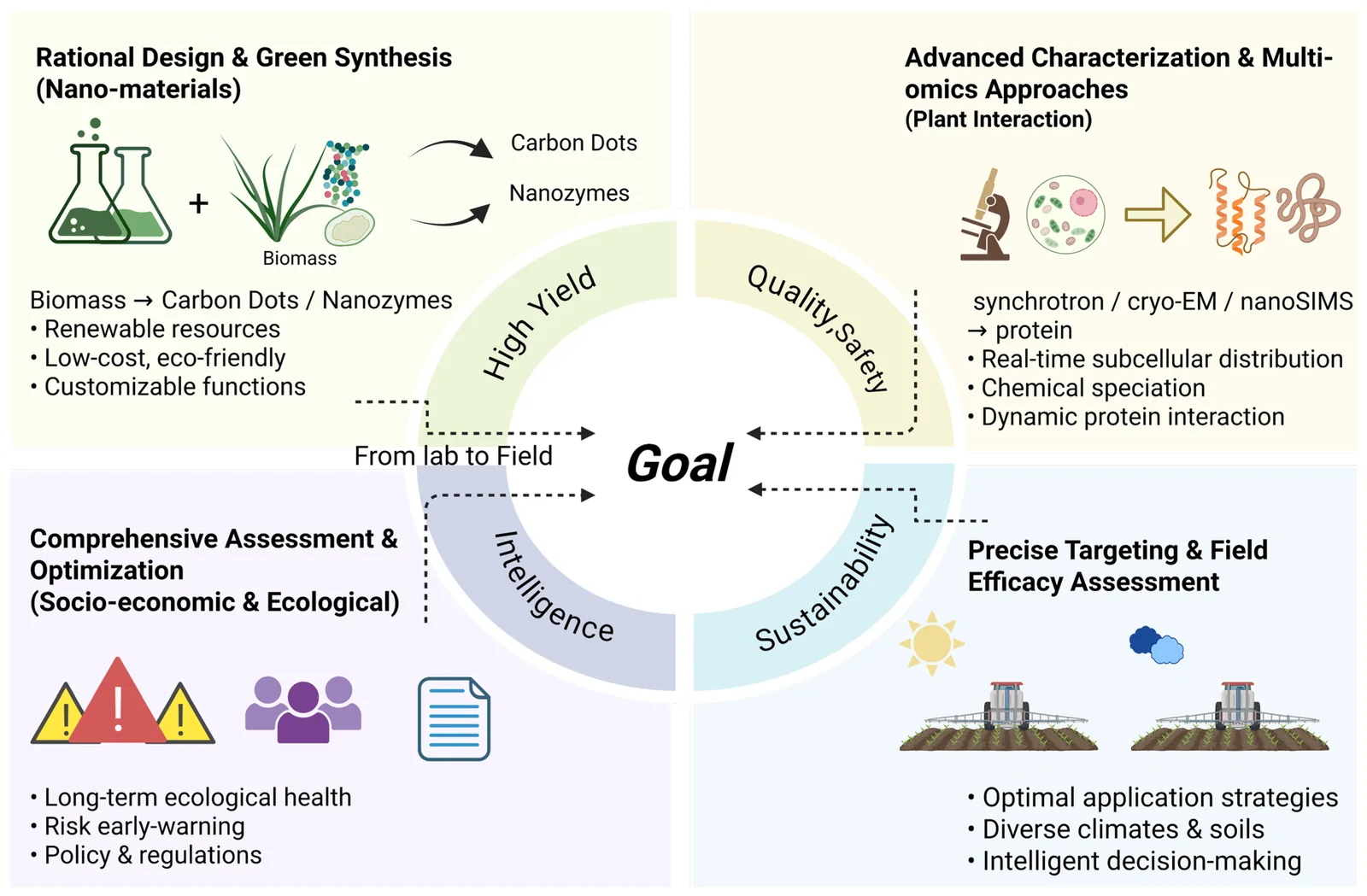
A closed-loop framework for nano-enabled crop photosynthesis: from lab to field. Closed-loop research and application system for nanomaterials supporting crop photosynthesis to improve yield and quality, from rational design and green synthesis, advanced characterization and multi-omics, comprehensive assessment and optimization, to precise targeting and field efficacy evaluation. Created in BioRender: https://BioRender.com/vjev3dm (accessed on 26 October 2026).

The prospects for translation are cautiously encouraging. Carbon dots and other photonic nanomaterials offer a mechanism for improving light utilization in crops, supported by in planta evidence in rice and other systems [10,11,43,72], and nanofertilizers promise improved nutrient-use efficiency and stress resilience that could complement conventional agronomy [33,34,108,110,128]. Selenium nanoparticles have been proposed as dual-function micronutrient carriers and redox modulators that improve crop stress resistance [131]. The barriers to adoption are not primarily technical: they include the lack of robust field-scale evidence, unresolved ecotoxicological and food-safety questions, and the absence of regulatory frameworks tailored to nano-enabled agricultural inputs [26,44,111]. We therefore recommend that the next phase of research be organized around hypothesis-driven, physiology-aware field trials in which photosynthetic type, dose–response structure, and long-term fate are measured as standard variables, and in which results are reported with sufficient detail to be combined into predictive models. If the field adopts this discipline, nano-engineered photosynthetic regulation can move from a catalog of promising anecdotes to an engineering discipline with defensible design rules (Figure 4).

## Figures and Tables

**Table 1:** Representative nanomaterial effects on photosynthesis and yield in C3 plants.

Nanomaterial Type	Application Method	Plant Species	Main Regulatory Mechanism	Photosynthetic/Yield Effect	References
Carbon dots (CDs)	Foliar spray	Rice(C3)	Raises Rubisco activity and leaf Pn; upregulates sucrose-transporter genes (*OsSUTs*, *OsSWEETs*) and stem/grain starch enzymes, enhancing stem-to-grain assimilate translocation under N deficiency	Pn ↑ (8.5–61.5%), Yield ↑ 12–13% (recovery under N deficiency)	[89]
Carbon dots (CDs)	Foliar spray	Tomato (C3)	Delays leaf senescence, maintains photosynthetic capacity	Photosynthate accumulation ↑, Yield ↑	[90]
Mesoporous carbon NPs	Hydroponic root exposure (0–150 mg/L, 20 d)	Rice (C3)	Concentration-dependent growth inhibition; altered endogenous hormones (brassinolide, IPA, DHZR); no chloroplast entry or electron-transport evidence	At 150 mg/L root/shoot length reduced up to ~70%/57% (size-dependent)	[93]
Nano-MoS_2_	Soil application	Soybean (C3)	Dynamic transformation, provides edge active sites, accelerates PSII electron transport	Photosynthetic rate ↑, Biomass ↑	[58]
CeO_2_ NPs	Soil amendment (10, 50, 100 mg/kg)	Radish (C3)	Promoted fresh-biomass accumulation (root expansion 2.2-fold at 50 mg/kg); relative chlorophyll +12.5–12.9%; Ce retained mainly in roots; antioxidant enzymes enhanced	Biomass up, chlorophyll up (promotional/hormetic at tested doses)	[25]
Ag NPs	Seed/foliar treatment	Rice (C3)	Generates mild ROS, primes stress/immune responses (“stress training”)	Stress resilience ↑, photosynthesis maintained under stress	[94]
TiO_2_ NPs	Foliar spray	Lettuce(C3)	Reprograms cellular metabolism and antioxidant defenses to mitigate UV-B-induced oxidative damage	Mitigates photosynthetic decline under UV-B (metabolic/antioxidant protection, not a direct light-reaction enhancement)	[69]
TiO_2_ NPs	Foliar spray	Fragrant rice (C3)	Regulates antioxidant response, increases photosynthetic pigments	Photosynthetic performance ↑	[15]
ZnO NPs	Foliar spray	Rice (C3)	Increases stomatal conductance and intercellular CO_2_, enhances carbonic anhydrase	Photosynthetic rate ↑, Yield ↑	[16]
ZnO NPs	Foliar spray	Tea plant (C3)	Enhances photosynthetic capacity, improves phyllosphere microbial community	New shoot growth ↑	[18]
Fe_3_O_4_ NPs	Foliar spray	Soybean (C3)	Enhances source–sink communication, optimizes carbon partitioning	Yield ↑	[21]
FeO NPs	Foliar spray	Peanut (C3)	Restores PSII maximum photochemical efficiency under salt stress	Photosynthetic performance ↑	[22]
Ag NPs	Aquatic exposure	*Spirodela polyrhiza* (C3, aquatic)	Inhibits NADP-dependent GAPDH enzyme activity	Photosynthetic rate ↓	[86]

Note: ↑ indicates an increase and ↓ indicates a decrease; Pn denotes net photosynthetic rate.

**Table 2:** Representative nano-fertilizer effects on crop yield and quality.

Nano-Fertilizer Type	Crop	Application Method	Yield Effect	Quality Improvement	Key Mechanism	References
Carbon dots (CDs)	Rice	Foliar spray	~10–15% increase (N-deficient)	Higher N use efficiency, grain weight	Enhanced photosynthesis, upregulated sucrose transport, remobilization	[89]
Carbon dots (CDs)	Tomato	Foliar spray	Significant increase in fruit weight and total yield	Delayed senescence, extended harvest	Maintenance of photosynthetic capacity	[90]
Zn-CD nanohybrid	Lettuce	Foliar spray	Significant increase in fresh/dry weight	Higher soluble sugar and ascorbic acid	Synergy of Zn nutrition and light conversion	[91]
Nano-MoS_2_	Soybean	Soil application	~30% higher grain yield than conventional Na_2_MoO_4_ molybdate (~35% vs. untreated control) at 10 mg/kg	Improved seed quality (protein trend)	Dynamic transformation releases Mo/S into N-metabolism and antioxidant enzymes; residual particles act as nanozymes; delayed nodule senescence	[58]
Nano-N-P-K	Wheat	Foliar/Soil	Significant grain yield increase	Higher protein content	Enhanced antioxidant system, balanced nutrition	[95]

**Table 3:** Direct head-to-head C3 vs. C4 comparisons under matched nanomaterial exposure.

Ref.	Material (Dose, Route)	C3 vs. C4 Species	Measured Endpoints	Observed C3 vs. C4 Response	Mechanism (Measured/Inferred)
[114]	La_2_O_3_ NPs, hydroponic 10 mg/L	Soybean vs. maize	Net Pn, Fv/Fm, PEPC, Rubisco	Both inhibited; Pn −8.77% soybean vs. −55.52% maize; PEPC/Rubisco suppressed only in maize	Measured: gas exchange, enzymes. Inferred: bundle-sheath access.
[28]	CuO, Ag, TiO_2_, MWCNT, FLG, soil-spiked	Wheat/rice vs. amaranth/maize	Chlorophyll, Fv/Fm, tissue metal, TEM	Rice chlorophyll −40.9 to −54.2% vs. maize −3.5 to −15.1%; Fv/Fm down up to 51% in rice, unchanged in amaranth/maize; C3 took up more metal	Measured: pigments, Fv/Fm, uptake, TEM. Inferred: bundle-sheath compartmentation.
[49]	In_2_O_3_ NPs, soil, ambient vs. elevated CO_2_	Barley vs. maize	Growth, photosynthesis, H_2_O_2_, P/Fe, SOD	In_2_O_3_ reduced growth/photosynthesis and impaired P/Fe; C3 barley more sensitive overall, C4 maize more responsive to elevated CO_2_; validated by FeSOD-overexpressing maize	Measured: biomass, Pn, ions, SOD, transgenic validation.
[115]	In_2_O_3_ NPs, soil, ambient vs. elevated CO_2_	Wheat vs. sorghum	Biomass, Pn, sugars, proline, polyamines	In_2_O_3_ retarded biomass/photosynthesis, especially young C3 leaves; elevated CO_2_ mitigated more strongly in C4 young leaves	Measured: biomass, Pn, metabolite profiling.
[116]	As_2_O_3_ NPs, soil, ambient vs. elevated CO_2_	Barley vs. maize	Growth, photosynthesis, H_2_O_2_, proline, anthocyanins	Barley accumulated more NPs and suffered more oxidative damage; elevated CO_2_ mitigated photosynthetic loss in both via species-specific pathways	Measured: physiology, primary/secondary metabolites.
[113]	Carbon quantum dots (empty-fruit-bunch biochar), foliar spray	Rice vs. corn	CO_2_ assimilation (Pn), stomatal conductance	Photosynthesis enhanced in both crops; C3/C4 response compared	Measured: gas exchange, stomatal conductance. Inferred: photon conversion.

## Data Availability

No new data were created or analyzed in this study. Data sharing is not applicable to this article.
